# Diversity and its causes: Lewontin on racism, biological determinism and the adaptationist programme

**DOI:** 10.1098/rstb.2020.0417

**Published:** 2022-06-06

**Authors:** Hao Shen, Marcus W. Feldman

**Affiliations:** Department of Biology, Stanford University, Stanford, CA 94305, USA

**Keywords:** racial classification, biological determinism, heritability, adaptationism

## Abstract

Lewontin's 1972 paper (RC Lewontin, 1972 *The apportionment of human diversity*, in *Evolutionary biology, vol. 6* (eds T Dobzhansky, MK Hecht, WC Steere), pp. 381–398) can be viewed as one foray in his battle against biological determinism. Our paper shows where Lewontin, *The apportionment of human diversity*, fits in the debate over human classification that it stimulated. We outline three assumptions inherent in the biological deterministic view of human phenotypic diversity and show how the 1972 paper, as well as Lewontin's papers in 1970 and 1974 on the problems with the heritability statistic and his 1979 criticism of naive pan-selectionism, invalidate these assumptions. These papers were crucial components of his campaign against biological determinism and the racism with which it was associated. In the current climate of widespread racism and the rise of sociogenomics, it is important to revisit Lewontin's writings and to disseminate the messages they contain.

This article is part of the theme issue ‘Celebrating 50 years since Lewontin's apportionment of human diversity’.

## 1. Introduction

Throughout his career, Lewontin wrote many important scientific papers as well as essays for the public on topics related to biology and evolution in which he addressed the relationship between biology and society. He fought against the trend of using biology to justify and strengthen the existing structural inequality among races, classes and sexes. His papers on human diversity and racial classification opened new research directions in human genetics, and his more popular writing, for example, his essays for the *New York Review*, some collected in Lewontin [1], also had a major impact on the public's understanding of limitations on the contribution that evolutionary biology might make to such social issues as racial differences. In this, the 50th year since Lewontin published his landmark 1972 paper, we undertake a retrospective review of a series of his important publications in an attempt to reconstruct his train of thought as he combatted biological racism.

In 1984, Lewontin, Steven Rose and Leon Kamin, published *Not In Our Genes: Biology, Ideology and Human Nature*. In this book (e.g. pp. 243–244), Lewontin and his colleagues summarized the logic of ‘sociobiology’, the theory that there exist universal aspects of human nature, which are genetically determined, and which were established by natural selection during evolutionary history. Although these biological deterministic ideas about human nature focus mainly on common human features, they have often been applied more generally to human diversity. For the traits that are viewed as ‘essential’, biological determinism assumes that among human groups the pattern of genetically determined human variation has been established by natural selection under different environments: as a result, socio-economic inequalities between races (classes) are natural and immutable (e.g. chapter 2 of [2]).

The biological deterministic view of human diversity can be regarded as being predicated on three crucial assumptions about genotype–phenotype relationships:

1. There exist typical patterns of human phenotypic variation that are strongly predicted by group membership and that are pervasive across many phenotypes;
2. These patterns of variation are mostly genetically determined;
3. Historically, observed patterns of human phenotypic variation are the result of natural selection on these phenotypes.

In the context of biological racism, the first assumption refers to the pattern exhibited by the phenotypes that are most commonly used to define races, such as skin colour, hair form and eye shape, for which the among-group diversity is larger than the within-group diversity. Biological determinists assume that a similar pattern exists for ‘essential’ human traits such as intelligence, educability or criminality. The other two assumptions concern the causation of the universal diversity pattern in assumption 1. Using the distinction made by Ernst Mayr [3], the second is about the ‘proximate cause’ of the pattern, while the third is about the ‘ultimate cause’ of the pattern.

Beginning in the early 1970s, Lewontin wrote a series of papers on racism, biological determinism and reductionism [4–10]. In retrospect these works form a systematic critique of the three assumptions above, although he did not frame his works in this way. For example, Lewontin [5] can be viewed as refuting the first assumption; in that paper, he showed that the diversity pattern exhibited by the phenotypes that are used to define race, and their corresponding genotypes, are far from universal (see [11], this volume). Lewontin [4] and [6] and Feldman and Lewontin [9] illustrated the methodological problems in human genetic studies that used the heritability statistic to legitimize the second assumption. Gould & Lewontin [10] attacked the adaptationist programme, the third assumption used by biological determinists. These papers, together with Lewontin [8], established a wholistic causative scheme in evolutionary biology that reflects the ontological complexity of the biological world.

In the following sections, we will review Lewontin's analyses and conclusions and show that his reasoning remains valid today. We also refer to other studies that confirm and develop Lewontin's thesis. Our aim is to recognize and build on Lewontin's important legacy, in scientific and popular forums, of strong opposition to both racism and biological determinism.

## 2. Lewontin and diversity

At the time Lewontin wrote his landmark paper, worldwide human data on polymorphic genetic loci were readily available for 17 blood groups and enzymes. Lewontin asked one statistical question, the answer to which occupies more than 17.5 pages of the paper, and he devoted 10 lines of text to his interpretation of the statistical results. The statistical question was: what fraction of worldwide diversity in single-locus allele frequencies was associated with the diversity among the seven ‘races’ he considered, or associated with diversity among populations assumed to be within a race, or associated with diversity over all the populations in the total sample. He found that the mean proportion of diversity that was ‘contained within populations’ was 85.4%, while that between races was 6.3%.

Lewontin [12] had published a table of 33 blood group heterozygosities in the English population. Nine of these blood groups are among the 17 markers in the 1972 paper. However, of the 33 in the 1967 paper, only eleven had English heterozygosities greater than 0.01. Although Harris [13] had published data on ten enzymes in human blood, the analysis in Lewontin [12] included data only on blood groups and then only up to 1962. However, at the end of the 1967 paper, Lewontin does compare the 30% of enzyme loci that Lewontin & Hubby [14] found to be polymorphic in *Drosophila pseudoobscura* with the three out of 10 enzymes in human blood that Harris [13] found to be polymorphic. Lewontin [5] tabulates the range of allele frequencies known for each of the 17 loci and the populations studied up to that time. Later comparisons of heterozygosities in worldwide populations were made in the context of human migration out of Africa and showed a strong signal of decreasing heterozygosity with distance from Africa (fig. 3B in [15]).

Since Lewontin [5], data on human polymorphisms at a larger number of classical (blood group and enzyme) polymorphisms, restriction fragment length polymorphisms (RFLPs), microsatellite (short tandem repeat) polymorphisms (STRs) and single nucleotide polymorphisms (SNPs) have accumulated. The monumental treatise by Cavalli-Sforza *et al*. [16] used the first two kinds of data to infer spatial and temporal patterns of human genetic evolution.

Cavalli-Sforza and his colleagues were the first to use microsatellite data in analyses that directly paralleled that of Lewontin [5]. Bowcock *et al*. [17] collected data on 30 microsatellite polymorphisms in 148 individuals from 14 populations representing five continental regions. They found the average allele-sharing distance among individuals within their 14 populations to be 0.64 compared to 0.73 for individuals from different continents, concluding ‘that the bulk of the genetic variation is within populations’. Barbujani *et al*. [18] analysed the 30 microsatellites from Bowcock *et al*. [17] together with 70 RFLPs, all from 1109 individuals on four continents. They found that 84.4% of the diversity was within populations and 10.8% was between continents, results that were very similar to Lewontin's (see [11], for more on these analyses).

The advent of the Human Genome Diversity Panel (HGDP-CEPH) [19] presented the opportunity to study a large number of populations with many more microsatellite markers. With 377 microsatellites and analysis similar to that of Barbujani *et al*. [18], Rosenberg *et al*. [20] found that 93.2% of the diversity was within populations and 4.3% was between major continental regions (see also [21]). Nine hundred and thirty-eight unrelated samples from the HGDP-CEPH panel were studied at 642 690 SNPs by Li *et al*. [15], and using the same diversity analysis as Rosenberg *et al*. [20] they found that 88.9% of the variance was within populations and 9.0% was among the seven geographical regions most appropriate to their data. The answer to Lewontin's statistical question, namely that the overwhelming fraction of single-locus frequency diversity is within populations, with something less than 10% associated with continental or ‘racial’ differences, has now been confirmed many times. Lewontin's conclusion was that ‘our perception of relatively large differences between races and subgroups … is indeed a biased perception’ and ‘based on randomly chosen genetic differences, human races and populations are remarkably similar to each other’ [5, p. 397]. Barbujani and Cavalli-Sforza [18, p. 4518] came to a similar conclusion: ‘… the burden of proof is now on the supporters of a biological basis for human racial classification’.

## 3. Critiques and resolution

Lewontin remarked [5, p. 397], ‘If we are to assess the meaning of racial classifications in genetic terms, we must concern ourselves with the usual racial divisions.’ He believed that racial classification was of ‘virtually no genetic or taxonomic significance’. However, controversy arose shortly after Lewontin published this paper. On the one hand, Nei & Roychoudhury [22,23] employed a similar single-locus approach and confirmed Lewontin's conclusion. On the other hand, Spielman and Smouse [24] and Smouse & Spielman [25], using multilocus combinations, suggested that the accumulation across loci of frequency differences could be used for classification, while Mitton [26] claimed that with multilocus combinations, the ratio of among-group variance to within-group variance could be significantly larger than found by Lewontin, who averaged over single loci. Mitton's claim was criticized by Lewontin [7] and others [27,28], and Mitton [29, p. 1143] responded that his multilocus approach might be very useful and ‘provide resolution of groups that is not apparent in a sequence of single-locus analyses’.

An important summary of this debate was made by Neel [30], who argued that the two sides were asking different questions. He concluded that Lewontin and Nei and colleagues were studying variation (diversity) partitioning by asking: ‘What proportion of all the genetic variation within some large group can be attributed to differences among subgroups and among individuals, on average, over all known loci?’ On the other hand, according to Neel, Spielman, Smouse and Mitton were studying classification, which asked ‘Are the levels of allelic frequency variation found between human populations sufficient to generate a useful taxonomy?’ This distinction was also emphasized by Edwards [31], who criticized Lewontin for seeming to reduce the classification problem to the diversity-partitioning problem, and (not knowing about Mitton's contribution) used a model similar to that of Mitton [26] to show that such a reduction was not correct.

Neel [30] and Edwards [31] made the important clarification that establishing an appropriate diversity measure and using it for partitioning diversity within and between groups is in general different from establishing a classification system for biological systematics [11,32]. The difference involves the distinction between levels of difference and levels of similarity. A simple example is shown in box 1. As validated by later research on ancestry, such a systematic classification is possible even if most of the diversity lies within groups. Was Lewontin wrong in saying that racial classification is of ‘virtually no genetic or taxonomic significance’, and were Mitton and Edwards correct? Our answer to both of these is ‘no’. If Mitton and Edwards were merely emphasizing the difference between the two problems and arguing that human genetic variation can be used to establish a useful taxonomy, we believe that Lewontin would agree, as expressed in Feldman & Lewontin [34]. However, Mitton and Edwards supported the potential utility of the classical race classification, and therein lies the true problem with their critique of Lewontin; they used the terms ‘race’ and ‘classification’ in a way that was different from the way Lewontin used them (box 1).

Box 1. **Toy model for difference versus similarity.**

An intuitive toy model to illustrate difference versus similarity would be the following. Assume that within a group, two randomly chosen individuals have 90% probability of having different genotypes, with 10% probability of having the same genotype, and two random individuals chosen from two different groups (or from the whole population, which will be similar if the number of groups is not too small) have 99% probability of having different genotypes and 1% probability of having the same genotype. Then individuals within the same group are almost as different as individuals from different groups. It is much more likely (although the probabilities are both very low) that two individuals from the same group have the same genotype than if they came from different groups. This information about similarity (if we use the similarity measure to indicate the probability of having the same genotype) can be used for ancestry in forensic studies. However, it would be inappropriate to use the compliment of similarity to show how different individuals are. For a more accurate mathematical understanding of the difference versus similarity problem, one can employ the Hedrick similarity measure [33] used in Mitton [26], and compare the compliment of this similarity measure with a diversity measure based on diversity partitioning.

Historically, ‘race’ in genetics literature has referred to people's continental origin but is more commonly understood in terms of some easily perceived phenotypes. The second definition of race is notorious for its political application, i.e. extrapolating from differences among races/groups in some observed phenotypes to essential human differences (such as in intelligence). However, ‘classification’ can mean both a classification system and the results of the classification. The former includes the latter, but also other components such as metaphysical presumptions of the classification (for example, all three assumptions mentioned earlier, especially the first assumption), the statistical methodology used in the classification, and the social consequences of the classification.

Mitton [29] and Edwards [31, p. 799] cited the foundational papers that introduced evolutionary trees into human population genetics [35–37]. By referring to the tree-like structures of the human population, it seems that for Mitton and Edwards, ‘race’ means continental origin, and ‘classification’ is just the result of the process of classification. The tree-like structure definitely entails more information than just continental origin. For example, Leslie *et al*. [38] found that their sample of 2039 British individuals could be divided into 17 subgroups according to their ancestry profiles based on SNPs. However, nobody would say that there are 17 races in Britain. Thus, the ‘race’ that Mitton and Edwards referred to, in the context of evolutionary trees, should be best understood at the level of continental origin.

It is true that small differences in allele frequencies at large numbers of markers, with appropriate algorithms [39–41], allow continental clustering [15,20,21]. For pairs of populations from different clusters, genetic distance is generally larger than between pairs of populations from the same clusters, indicating that the history of migration is important [34,42,43]. However, for Lewontin, ‘racial classification’ had a totally different meaning from continental origin. For him, ‘race’ meant the classical race typology ‘requiring small numbers of genetic characters with dramatic biologically significant differences across groups to be typical of the genome-wide pattern’ [32], and ‘classification’ means the ‘classification system’. This is why Lewontin emphasized that genes that code for skin colour, hair form and eye shape, which are the phenotypes most commonly used to define races, are atypical of the genome in general. He did not argue from diversity partitioning to classification; instead, he stressed that the traditional racial classification relies on certain assumptions about diversity partitioning (assumption 1). As a critique of the traditional racial classification system, Lewontin used the diversity partitioning analysis for a legitimate attack on the hidden assumptions of that system and its flawed methodology. Thus, we can say that a statistical fallacy did exist, and the distinction is important, but Lewontin did not commit that fallacy and calling his conclusion a fallacy is erroneous. The logic underlying our claim here is shown in box 2.

Box 2. **Justification of classification.**

The traditional classification of humans into races entails two things: first, the existence of high between-race variation; and second, the classification, should, at least partly, coincide with continental origin because of the large variation between populations from different continents. Lewontin showed that high between-race diversity seen for some phenotypes was not typical of genetic variation, and thus the justification for a biology-based classification was problematic. This might not mean that the classification was wrong, since a problem with the reasons for the classification does not necessarily mean that the classification itself is wrong. (It is, however, important to emphasize that the racial classification was also wrong, although the final proof of this was provided by later studies of genomic variation showing widespread mixed ancestry, about which Lewontin could not have known in 1972.) It seems that Edwards understood ‘classification’ as merely the result of the process of division into races. From his point of view, Lewontin was trying to falsify the classification by attacking the justification for it. However, for Lewontin, classification meant the system of classification, and this system, whose justification was methodologically problematic, should be criticized, which is exactly what his 1972 paper accomplished.

It is important to note that the two definitions of ‘race’, i.e. race based on continental origin and the traditional definition based on a small number of genotype–phenotype pairs, as shown by the data, only partially coincide with each other (e.g. [34]). Additionally, the term ‘continental origin’ is now much more complex than simply where an individual comes from, and should be replaced by ‘ancestry profile’. Modern data show that mixed ancestry is widespread, which is definitely beyond the scope of the traditional conception of race. Understanding ‘ancestry profile’ and ‘mixed ancestry’ entails understanding the statistical methods used in Pritchard *et al*. [39], Alexander *et al*. [41], Bryc *et al*. [44], Lawson *et al*. [45] and Mallick *et al*. [46]. The wide methodological gap between today's ancestry analysis and traditional racial classification actually debunks the classical racial classification system. That is to say, even if ancestry analysis and the traditional racial classification were to give exactly the same results, which of course they do not, the fact that they have totally different model assumptions and methodology shows how problematic the traditional racial classification system is (see box 2). As pointed out by Feldman & Lewontin [34, p. 99], ‘Lines of ancestry of an individual can provide information on the likelihood that the person carries certain alleles. Lines of ancestry, rather than arbitrary racial categories, can provide much accurate, biologically interesting and potentially medically useful information.’

We conclude that Mitton and Edwards' critique of Lewontin [5] missed the point. The partial coincidence between the results of the essentialist biological racial classification and continental origin cannot justify the methodological legitimacy of the former. Lewontin's paper [5] not only quantified diversity partitioning but also constituted a scientific negation of racial classification, as long as we understand racial classification in its historical context. As pointed out by Rosenberg [32, p. 401], ‘The destructive typological race theories of the past, requiring small numbers of genetic characters with dramatic biologically significant differences across groups to be typical of the genome-wide pattern, are clearly belied by the high within-population variance component’ (see also [47]).

## 4. Lewontin on heritability

As mentioned in the introduction, Lewontin [5] can be viewed as a general critique of the first assumption. This and later studies showed that for any single gene and its functions, the diversity pattern exhibited by the phenotypes, and their corresponding genotypes that are used to define race, are far from universal. For most phenotypes paired with a single gene, the within-group diversity will be much larger than the among-group diversity rather than vice versa. (In principle we should distinguish between genetic and phenotypic differentiation due to the existence of dominance. However, in the single gene case, the within-group genetic phenotypic diversities should be similar when the among-group genetic diversity is very small.)

Lewontin's analysis did not refer to phenotype-genotype relationships where the phenotype is a complex trait that is possibly associated with many genes. This gap was partly filled by later research, for example by Edge & Rosenberg [48], on the relationship between genetic differentiation and phenotypic differentiation, which showed that for selectively neutral, additive and completely heritable quantitative traits, phenotypic differentiation was not strongly influenced by the number of loci and would be roughly equal to the genetic differentiation at a single neutral locus. Of course, in real life the relationship between genetic and phenotypic differentiation is rather complex, but it is fair to say that it would be unreasonable to use the pattern of variation exhibited by traits such as skin colour as the default prediction. Analyses of genetic and phenotypic differentiation do not exclude the possibility that some traits (possibly viewed as ‘essential’) exhibit a similar pattern to that of skin colour. However, they do suggest that evidence is needed to validate such a hypothesis. Further, even if the pattern proposed in the first assumption above (i.e. large among-group variation compared to within-group variation) were observed, additional evidence would still be needed for the second assumption, i.e. that trait diversity is genetically determined.

For many complex traits (such as intelligence), we know very little about any associated genes. Thus the assumptions, especially the second assumption above, cannot be tested directly (this is still as true today as it was in the 1970s). Ironically, despite the extreme paucity of evidence, genetic determinists in the 1970s were still confident enough to assert a deterministic relationship between complex traits and genes that were not known. A typical example is Arthur Jensen, an educational psychologist who claimed that differences in IQ (a trait used to represent intelligence) between African-Americans and European Americans were largely caused by genetic differences [49]. Jensen's racist position echoes the eugenic position that pervaded natural and social sciences in the first half of the twentieth century, and the statistic upon which Jensen's claim (and that of his contemporary, extreme proponent of genetic essentialism in racial differences, William Shockley) was based is heritability.

Heritability, originally used in agriculture to predict the success of animal breeding plans, is the fraction of phenotypic variation that is associated with the genetic term in a linear model where the phenotype is the sum of genetic and environmental terms. Heritability became widely used in behaviour genetics during the second half of the twentieth century to represent the extent to which the variation of a trait was ‘genetically determined,’ which, as Lewontin [6] pointed out, is deeply problematic (see also [9]). From the high (estimated) heritability of IQ within people of European ancestry, Jensen made the erroneous inference that IQ differences between Whites and African-Americans were due to genetic differences between these two groups. The inference that the high heritability of IQ entails that it is largely determined by genes became ubiquitous in behaviour genetics in the second half of the twentieth century. For example, based on the genetic determination of intelligence inferred from the heritability of IQ, Herrnstein & Murray [50] in their widely read book *The Bell Curve* proposed policies related to components of society and its governance.

Lewontin [4, p. 7] attacked Jensen's [49] statement that ‘genetic factors are strongly implicated in the average negro-white intelligence difference’. Although Jensen shrouded his statement in what Lewontin called ‘academic disclaimers', what Jensen really claimed was ‘most of the difference in IQ between blacks and whites is genetical’ [4, p. 7]. Jensen's conclusions were based on the high heritability of IQ in white populations, and his inference of genetic causation is incorrect: ‘Genetic basis of the difference between two populations bears no logical or empirical relation to the heritability within populations and cannot be inferred from it’ [4, p. 7]. Lewontin then gave conceptual examples from plant biology that illustrate the fallacy of Jensen's inference. In the first example, each of two populations is highly inbred and contains no genetic variation, but the two populations do differ genetically. Within these populations, the variance for a measured trait will be entirely environmental, but the difference in average value of that trait between the populations is entirely genetic despite the heritability within each population being zero. In the second example, variation within populations is entirely genetic, so that the heritability of a trait is 100%. However, the difference between the populations in the average value of that trait is entirely environmental.

In this paper, Lewontin was attacking Jensen's attribution of what he claimed was the difference in educability between blacks and whites (as measured by the IQ difference between them) to genetic causes. Lewontin [6] subsequently addressed a more general issue of the inference of causation from analyses of variance, not just the case of heritability that Lewontin felt had to be addressed in view of Jensen's spurious claims.

Lewontin [6] expanded his criticism of the misuse of heritability to infer causal relationships between genotypes and phenotypes. He wrote that the partition of total phenotypic variance into genetic and environmental components depends upon ‘the actual distribution of genotypes and environments in the particular population sampled’ [6, p. 403]. The problem then is what should the canonical distribution of genotypes and environments be in the variance analysis. Should it be a uniform distribution of genotypes and environments or an arbitrary distribution chosen according to the researcher's interests, or a distribution that records historical forces acting on genotype frequencies and the ‘actual structure of the environments in which the population finds itself’? There could be completely different distributions for the same set of genotypes and environments. Heritability is spatio-temporally restricted; one cannot rely on the heritability of a certain trait since it always contains spatio-temporal information over and above the functional relations that constitute the reaction norm. Lewontin writes that although the model of the phenotype as the sum of genetic and environmental terms, with the heritability representing the genetic causation of the phenotype, is widely used, this process is mistaken because ‘the amount of environmental variance that appears depends on the genotypic distribution while the amount of genetic variance depends on the environmental distribution. Thus the appearance of separate causes is an illusion’ [6, p. 406]. He concludes that ‘the outcome of variance analysis … is not a tool for the elucidation of functional biological relations’ [6, p. 408].

Thus heritability is too specific since it is spatio-temporally restricted. Later research showed that it is also too general in that ‘it confounds different causative schemes in the same outcome’ [6, p. 403]. It was well known that assortative mating [51] and population structure [52] could inflate estimates of heritability. Also vertical cultural transmission [53–57] could result in misleadingly high estimates of heritability. With vertical cultural transmission, the causative meaning of heritability, even in the spatiotemorally restricted sense, will be very dubious [58]. These studies agreed with Lewontin [6] that heritability analysis was not a good tool for causal research in human genetics. Heritability is still used today in genome-wide association studies and behaviour genetics (e.g. [59]); however, the critique by Lewontin, that it is both too specific and too general remains a fundamental problem for researchers who seek causative explanations from heritability analyses or polygenic risk scores. For example, the population specificity of polygenic risk scores has been shown recently by Privé *et al*. [60].

Eight years after his paper on variance and causation, Lewontin and his Ph.D. student Anand Gupta published the results of their experiments (done in 1978) on norms of reaction in *Drosophila pseudoobscura* [61]. They showed that the sternite bristle numbers of different genotypic strains were sensitive to the temperature at which the flies were raised. Different strains showed different effects of changes in the temperature. The paper concludes (p. 948) with a statement relevant to both variance/causal analysis, and the process of natural selection:

Without knowing the norms of reaction, the present distribution of environments, the present distribution of genotypes, and without then specifying which environments and which genotypes are to be fixed or eliminated, it is impossible to predict whether the total variation would be increased, decreased, or remain unchanged by environmental or genetic changes, or what the outcome of natural selection would be.

## 5. Lewontin on the adaptationist programme and construction

We have seen how Lewontin criticized the second assumption of biological determinism. As mentioned above, biological determinists not only argued that human essential phenotypic differences were genetically determined but suggested that such differences were established by natural selection. This radical claim can be viewed as an extension of their belief in the universality of genetic causation of phenotypic differences among human groups from the proximate to the ultimate levels [3]. This claim suffers from obvious weakness; for example, if the genotype–phenotype relationship is unknown, and the genetic basis of certain traits may not exist at all, to what extent is such an inference different from storytelling? Apart from the semantic problem (that ‘traits’ are artificially defined—a typical example is IQ), this theory shares the characteristics of what Lewontin called the ‘adaptationist programme’ [10, p. 581], which ‘proceeds by breaking an organism into unitary ‘traits’ and proposing an adaptive story for each considered separately’. Regarding human diversity, we highlight two important points made by Gould & Lewontin.

First, even for a trait that is entirely determined by genetics—for example, one caused by a single gene—it may not be appropriate to view natural selection as the exclusive explanation of genetic variation at that locus within or among groups. As Gould & Lewontin [10, p. 590] point out, ‘The stochastic process of change in gene frequency by random genetic drift … has several important consequences. First, populations and species will become genetically differentiated and even fixed for different alleles and a locus in the complete absence of any selective force at all.’ It is important to note that almost all microsatellite and SNP loci used in ancestry analysis are selectively neutral, and observed population structure is more likely to reflect migration history, founder effects and genetic drift than natural selection [43]. Lewontin did not reject natural selection as an important evolutionary force. Actually, he proposed a statistical test for natural selection, the Lewontin–Krakauer test [62]. An interesting fact related to Lewontin [5] and Lewontin & Krakauer [62] is that the traits in Lewontin [5], such as blood groups, might be significant according to the Lewontin–Krakauer test and turn out to be under selection, while the pattern Lewontin discovered in the 1972 paper (within-group diversity being much larger than between-group diversity) could remain. However, among the genetically determined traits that are under selection, the patterns exhibited by phenotypes that are most commonly used to define races, such as skin colour, might be quite extreme; it would, therefore, be unreasonable to assume that these phenotypes are representative of general patterns of variation. What Lewontin was against was viewing natural selection as the default or even the only plausible explanation of phenotypic variation.

Second, as with Lewontin's [6] admonition against the nature/nurture dichotomous approach to explaining the causes of the phenotype differences (the appearance of separation of causes is a pure illusion), Gould & Lewontin [10] argued against looking for selection on specific bits of phenotype because of the connections between all the bits. The adaptationist programme, they claimed, was focused on traits rather than constraints and modes of development. Just as the norm of reaction approach entails that the phenotype is the joint product of genes and environment, we should not focus on separate bits of phenotype ‘for if selection can break any correlation and optimize parts separately then an organism's integration counts for little.’ Instead we should focus on ‘integrated developmental blocks and pervasive constraints of history and architecture’ [10, p. 597].

In 1983, Lewontin developed this holistic approach further [8]. Whereas Lewontin argued in 1974 against the possibility of separating genetic and environmental causes via analysis of variance, this was mainly a methodological critique and focused mainly on the causal path from environment to organism (phenotype) [6], his 1983 paper deepened the ontological aspect of this critique and extended to the causal path from the organism to environment. In the paper, he argued that organisms and environments interact in a process of coevolution: ‘Organisms do not adapt to their environments; they construct them out of the bits and pieces of the external world’ [8, p. 280] and proposed his famous pair of differential equations,

$$\frac{dO}{dt}=f(O,\;E)$$

and

$$\frac{dE}{dt}=g(O,\;E),$$

which describe the mutual effects of organism and environment upon each other, refuting the classic paradigm, which does not have *O* in the second differential equation. It is important to point out Lewontin did not deny the progress made in evolutionary ecology under such a paradigm during the second half of the twentieth century, since as was pointed out in his later essay, ‘Without such a separation of forces [referring to the omission of *O* from the second equation], the progress made by modern reductionist biology would have been impossible.’ However, Lewontin did believe it was time to change: ‘Yet for the scientific problems of today, that separation is bad biology and presents a barrier to further progress' [63, p. 31].

We can see that Lewontin's [6] focus on norms of reaction had been developing much earlier, as had his position on the duality of genotype and environment in the evolutionary process. In connection with his paper on construction [8], it is very interesting to recall two sentences from the 1955 paper [64, pp. 40–41]: ‘The existence of an optimal intermediate density indicates some facilitation between individuals of like genotypes.’ And the last sentence of Lewontin [64]: ‘It has been shown that facilitation may lead to a stable polymorphism of genotypes.’ Surely this line of thinking, derived from the experimental observations, presages his later emphasis on the organism as a key component of the evolutionary processes on that organism, i.e. niche construction.

Lewontin's [8] constructionist invocation inspired others, including Odling-Smee [65], who developed the above equations into a formal theory of niche construction [66]. The idea behind Lewontin's pair of equations is now an important aspect of modern evolutionary ecology and can be regarded as a continuation of his fight against the biological deterministic view of human existence. In the critique of sociobiology in *Not in Our Genes*, Lewontin and colleagues pointed out: ‘There is a more fundamental problem for biological human nature theories. Suppose that developmental biology were to reach the point where the developmental response to the environment of specific human genotypes could be specified with respect to behaviour. Under these circumstances, the characteristics of an individual could be predicted given the environment. But the environment is a social environment. What is it that determines the social environment? Somehow the characteristics of individuals are relevant, although they are not deterministic. … The laws of relation of individual genotype to phenotype cannot by themselves provide the laws of the development of society’ [2, p. 257].

In the 1970s, in addition to his work on the apportionment of genetic diversity and the misuse of the heritability statistic, Lewontin was deeply involved in the selection-neutrality debate that consumed many population geneticists during that period. Lewontin's approach to this decades-long debate was informed by his philosophical analysis of gene–environment interaction, his early experimental measures of fitness, and his mathematical and computational work on multi-locus population genetic models. Much of Lewontin's beautifully written textbook on population genetics, *The Genetic Basis of Evolutionary Change* [67], is devoted to his views on the debate, which, in common with many other population geneticists, he called the ‘balance’ (selection) versus the ‘classical’ (neutral) debate.

The difficulties of measuring the direction and strength of natural selection in nature are discussed in great detail in Chapter 5 of Lewontin [67]. Lewontin's conclusion, after summarizing the data on genetic variation, was:

To the present moment no one has succeeded in measuring with any accuracy the net fitnesses of genotypes for any locus in any species in any environment in nature.

Earlier in his career, Lewontin had explored the forms that natural selection could take in controlled laboratory settings. His experiments with *Drosophila melanogaster* [64] showed that fitness, measured as survival rates of different genotypes, depended on the density of flies in their containers, with survival being highest at intermediate densities. Survival also depended on the mixture of genotypes that comprised the experimental population. He came to the general conclusion:

The viability of a genotype is a function of the other genotypes which coexist with it, the result of any particular combination not being predictable on the basis of the viability of the coexisting genotypes when tested in isolation. [64, p. 41]

Subsequent experiments with *Drosophila busckii* [68, p. 277] led to three important conclusions about the process of natural selection: (1) ‘It is not possible to make general statements about the relative viabilities of genotypes from a knowledge of those viabilities at a particular density’; (2) ‘The relative fitness of genotypes change as the frequencies of these genotypes change so that the course of natural selection cannot be predicted without a knowledge of the full norm of reaction of the genotypes with respect to density and competition’; (3) ‘Natural selection does not assure that the fitness of the population as a whole will be increased’.

The classical school derives from H. J. Muller, who assumed the pattern of genetic variation in nature (including humans) would reflect the difference between mutants and wild types that he saw in his laboratory. This would make most genetic variants deleterious, in which case there should be very little variation in naturally occurring species (including humans). The balance theory, as enunciated by Dobzhansky and his students, assumed that in nature, most genes would be polymorphic so ‘there would be immense genetic variation available for adaptation through natural selection [67, p. 30]. As we have seen with his apportionment of diversity [5] and the meaning of heritability [6], Lewontin [67] saw societal implications for these positions: ‘Muller believed in a genetic elite’ whose ‘superior genotypes’ would be ‘manifested in their superior behavioural phenotypes’. On the other hand, the balance school saw ‘human society as dependent for its functioning on the existence of a variety of genotypes, no one of which is absolutely superior to any other. Both schools are equally “biologistic” in that they believe the nature of human society to be strongly influenced by the distribution of genotypes in the species.’ Lewontin goes on, ‘Neither view admits the possibility that genetic variation is irrelevant to the present and future structure of human institutions, that the unique feature of man's biological nature is that he is not constrained by it’ [67, pp. 30–31].

When Lewontin writes that genetic variation is irrelevant to human institutions, this should be understood in the causal sense; correlation might exist between genetic variation and human institutions due to confounding or selection bias, but human institutions are not and should not be determined by genetic variation. Lewontin admits the existence of some biological constraints; for example, most peoples' height is between one and two metres, but humans can create airplanes, which overcomes this constraint. The extent to which the biological nature of humans is essential and unchangeable depends on the human's ability to modify itself (e.g. with medication) and the environment (both physical and social).

In a reprise to the ten concluding lines of interpretation in his 1972 paper, Lewontin [67, p. 156] concludes his long chapter 3 on genetic variation in natural populations with:

The taxonomic division of the human species into races places a disproportionate emphasis on a very small fraction of the total of human diversity. That scientists and non-scientists nevertheless continue to emphasize these genetically minor differences and find new ‘scientific’ justifications for doing so is an indication of the power of socioeconomically based ideology over the supposed objectivity of knowledge.

Indeed the whole history of the problem of genetic variation is a vivid illustration of the role that deeply embedded ideological assumptions play in determining scientific ‘truth’ and the direction of scientific inquiry. Those who, like Monod (1971), think that facts speak for themselves will suppose that the struggle between the classical and balanced schools is over, having been decisively concluded by the hard observations of the new molecular population genetics. But they will be wrong. The classical hypothesis has been developed in extended form, feeding upon, digesting, assimilating, and waxing fat on the very facts that were meant to give it fatal indigestion. It is not the facts but a world-view that is at issue, a divergence between those who, on the one hand, see the dynamical processes in populations as essentially conservative, purifying and protecting an adapted and rational status quo from the nonadaptive, corrupting, and irrational forces of random mutation, and those, on the other, for whom nature is process, and every existing order is unstable in the long run, who see as did Denis Diderot that, ‘Tout change, tout passe, il n'y a que le tout qui reste’ [everything changes, all things pass, only the totality remains] (see also Levins and Lewontin [69, p. 11]).

In the last chapter of the 1974 book, Lewontin focuses on the population genetic theory of multiple linked loci. This chapter should be understood as an amplification of Franklin and Lewontin [70], which showed that the theory of selection on single genes was grossly inadequate to describe epistatic selection on many linked genes: the units of selection should be combinations of haplotypes, not single-locus genotypes. There is here a remarkable confluence of the admonition in Gould & Lewontin ([10], referred to above) against the focus on ‘separate bits of phenotype’ and for a focus on ‘integrated developmental blocks' with what Lewontin [67, p. 318] wrote about single and multiple genes:

The fitness at a single locus ripped from its interactive context is about as relevant to real problems of evolutionary genetics as the study of the psychology of individuals isolated from their social context is to an understanding of man's sociopolitical evolution. In both cases context and interaction are not simply second-order effects to be superimposed on a primary monadic analysis. Context and interaction are of the essence.

Context and interaction are also central to Lewontin's mathematical work on the roles of epistasis and linkage in evolutionary genetics. His empirical study with M. J. D. White on association between inversion polymorphisms in the Australian grasshopper *Moraba scurra* [71] can be viewed as the stimulus behind his foundational 1960 paper that introduced the symmetric viability model of multilocus selection [72]. In this paper, the term ‘linkage disequilibrium’ was introduced, and the interaction between the rate of recombination and the form and strength of selection in producing stable association between genotype frequencies at two loci, association measured by linkage disequilibrium, was calculated explicitly.

Lewontin [73,74] took these mathematical findings further with computational investigations of models with five genetic loci under different assumptions on the form of natural selection. This multilocus analysis culminated in the massive computational analysis of 36 loci carried out by Franklin and Lewontin [70]. Comparing the 1964 conclusions with those of 1970, Franklin and Lewontin wrote:

The early finding was that loci far apart on the chromosome are held out of linkage equilibrium with each other by loci between them on the map. This is a result to be expected from the simplest ideas of correlation. The phenomenon explored in the present paper is quite different. Here, two adjacent loci are held in much higher correlation when embedded in a chromosome containing other loci interacting with them, than when they are considered in isolation. Such a result does not follow from simple considerations of correlation and arises from higher-order interactions that do not exist in the two-locus case. [70, p. 708]

Foreshadowing the subsequent massive expansion of computational genomics, Franklin and Lewontin [70, p. 734] concluded that it is possible ‘to frame a theory of population genetics which does not contain individual loci explicitly, but deals only with whole chromosomes, their recombination properties, and the effect of homozygosity of segments of various length. Such a theory is more consonant with the observations possible in population genetics than a theory framed in terms of gene frequencies.’

## 6. Conclusion

From Lewontin [5] to Lewontin [6] to Gould & Lewontin [10] to Lewontin [8] there is a thread: the common inferences and patterns of thought concerning biological determinism, in the era when these fundamental papers were written, were too simplistic.

Easily identified genotype–phenotype relations and the corresponding genomic population structure are far from universal; variance partitioning in heritability analysis is spatio-temporally restricted and causally dubious; breaking organisms into separate parts each under selection fails to explain the evolution of the whole organism; the separation of environment from organism ignores the process of niche construction. Lewontin showed that biological determinism systematically underestimates the complexity of the world and should be replaced by a different world view with clearer semantics, more rigorous methodology and more holistic ontology.

We can speculate that were Lewontin active today the explosion of genome-wide association studies (GWAS) and the concomitant calculations of polygenic risk scores for any measurable phenotype would have elicited even more of his inimitable admonitions against the dangers of unwarranted inference about causality. Moreover, his emphasis on the limitation of heritability statistic to a specific population in a specific environment becomes even more cogent as recent studies are demonstrating the poor portability of polygenic risk scores across different populations even within the same continent [60,75]. Scholars of genetics and evolutionary biology can still benefit greatly from Lewontin's scientific insights, as well as the power and elegance of the way he brought these insights to the attention of colleagues and the public.

## Data Availability

This article has no additional data.
